# *Escherichia coli* DNA repair helicase Lhr is also a uracil‐DNA glycosylase

**DOI:** 10.1111/mmi.15123

**Published:** 2023-07-14

**Authors:** Ryan J. Buckley, Anna Lou‐Hing, Karl M. Hanson, Nadia R. Ahmed, Christopher D. O. Cooper, Edward L. Bolt

**Affiliations:** ^1^ School of Life Sciences University of Nottingham Nottingham UK; ^2^ School of Biological and Geographical Sciences, School of Applied Sciences University of Huddersfield Huddersfield UK; ^3^ CHARM Therapeutics Ltd B900 Babraham Research Campus Cambridge UK

**Keywords:** DNA repair, DNA replication, glycosylase, helicase, uracil

## Abstract

DNA glycosylases protect genetic fidelity during DNA replication by removing potentially mutagenic chemically damaged DNA bases. Bacterial Lhr proteins are well‐characterized DNA repair helicases that are fused to additional 600–700 amino acids of unknown function, but with structural homology to SecB chaperones and AlkZ DNA glycosylases. Here, we identify that *Escherichia coli* Lhr is a uracil‐DNA glycosylase (UDG) that depends on an active site aspartic acid residue. We show that the Lhr DNA helicase activity is functionally independent of the UDG activity, but that the helicase domains are required for fully active UDG activity. Consistent with UDG activity, deletion of *lhr* from the *E. coli* chromosome sensitized cells to oxidative stress that triggers cytosine deamination to uracil. The ability of Lhr to translocate single‐stranded DNA and remove uracil bases suggests a surveillance role to seek and remove potentially mutagenic base changes during replication stress.

## Graphical abstract

Bacterial *L*arge *h*elicase‐*r*elated (Lhr) DNA repair proteins have well‐characterized helicase domains that translocate DNA, but in addition possess large (800 amino acid) C‐terminal domains of unknown function, which we show have uracil‐DNA glycosylase activity. We identify a novel active site for this function, and that loss of Lhr in *Escherichia coli* sensitizes cells to oxidative stress.

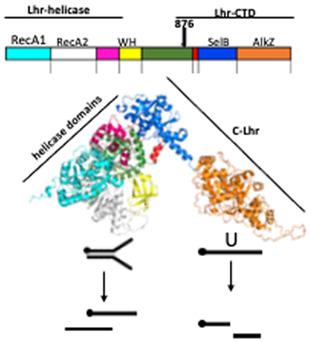

## 1 INTRODUCTION

Lhr (*L*arge *h*elicase*‐r*elated) proteins are ATP‐dependent 3′ to 5′ DNA translocases within the Superfamily 2 helicases (Hajj et al., 2021). The founder member of Lhr proteins was identified in bacteria (Reuven et al., 1995), and subsequently Lhr was found to be widely distributed across all clades of archaea (Chamieh et al., 2016). High amino acid sequence identity (typically about 30%) between archaeal and bacterial Lhr proteins is limited to 800–900 amino acids that form helicase domains from the Lhr N‐terminus—called the ‘Lhr‐Core’ (Ejaz et al., 2019). Biochemical analyses of the Lhr‐Core from the bacteria *Mycobacterium smegmatis* and *Pseudomonas putida* and from the archaeon *Methanothermobacter thermautotrophicus* have characterized Lhr translocation and helicase mechanism (Ejaz et al., 2018; Ejaz & Shuman, 2018; Ordonez & Shuman, 2013), and crystal structures of Lhr‐Cores highlight similarities with translocation by the archaeal DNA repair helicase Hel308 (Buttner et al., 2007; Ejaz et al., 2018), especially in interactions between their winged helix and RecA‐like domains (Johnson & Jackson, 2013; Northall et al., 2017).

In addition to the Lhr‐Core, bacterial Lhr proteins extend to 1400–1600 amino acids, in a C‐terminal protein region of unknown function, called Lhr‐CTD (Lhr‐C‐terminal domains). Structural modeling of the bacterial Lhr‐CTD (Buckley et al., 2020) and a subsequent cryo‐EM structure (Warren et al., 2021) provided intriguing clues to Lhr‐CTD function, including the presence of an array of tandem winged helix domains characteristic of the HTH_42 superfamily of proteins that have structural homology to the DNA glycosylase AlkZ (Buckley et al., 2020). Genetic analyses of the effects on bacterial and archaeal cells of deleting the *lhr* gene revealed mild sensitivities to agents that cause replication stress—UV irradiation (van Wolferen et al., 2015) and azidothymidine (AZT) (Cooper et al., 2015)—and transcriptional upregulation of *lhr* in response to mitomycin C (Rand et al., 2003). In this work, we report new insights about how Lhr contributes to DNA repair in bacteria. We demonstrate that the *Escherichia coli* Lhr protein has uracil‐DNA glycosylase (UDG) activity, in addition to its well‐characterized ATP‐dependent DNA translocase functions, and that cells lacking Lhr are sensitive to oxidative stress.

## 2 RESULTS

### 2.1 *E. coli* Lhr is an uracil‐DNA glycosylase requiring an active site aspartate

**FIGURE 1:**
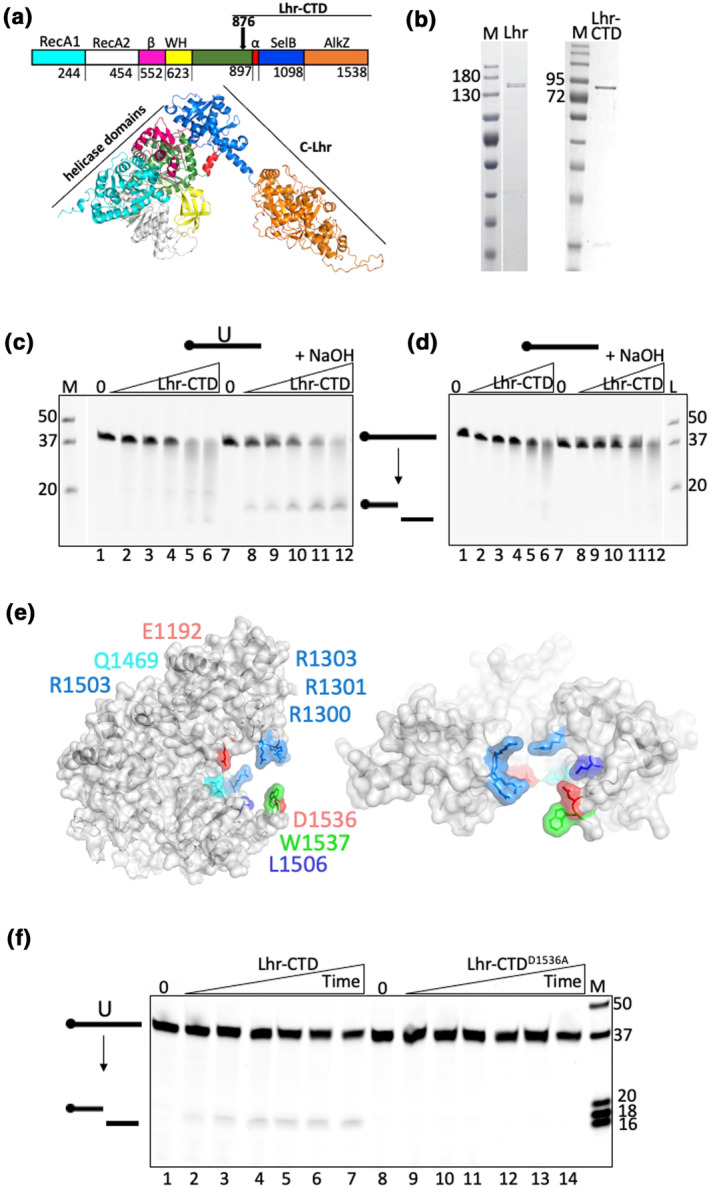
The *E. coli* Lhr‐CTD is a uracil‐DNA glycosylase requiring a catalytic aspartic acid. (a) AlphaFold 2 structural model of *E. coli* Lhr that is based on strong homology with the cryo‐EM structure of Lhr helicase core and Lhr‐CTD from *M. smegmatis*, respectively, PDB: 5V9X and PDB:7LHL. The *E. coli* Lhr‐Core helicase (amino acids 1–897) contains RecA domains, a beta‐sheet bundle (β), and a winged helix domain (WH) as indicated. Lhr‐CTD (amino acids 898–1538) comprises folds with structural homology to SecB chaperones and AlkZ glycosylases, as indicated. (b) Coomassie‐stained SDS‐PAGE acrylamide gels showing purified Lhr and Lhr‐CTD, with molecular mass ladder (M) values in kDa. (c) Products from mixing Lhr‐CTD (50, 100, 200, 400, and 800 nM) with 5′ Cy5‐ssDNA (12.5 nM) containing a d‐uracil base located 18 nucleotides from the fluorescent moiety as indicated (lanes 1–12), seen in a 15% denaturing acrylamide TBE gel. Addition of NaOH (lanes 8–12) causes β/δ elimination at the site of the abasic DNA product, resulting in DNA backbone cleavage. This confirms glycosylase protein activity. Marker (M) is made from known lengths of 5′ Cy5 ssDNA. (d) As for (c) in reactions containing unmodified 5′ Cy5‐ssDNA (12.5 nM). (e) Phyre2 structural model of *E. coli* Lhr‐CTD with predicted active site residues as labeled, including Lhr‐CTD residue Asp‐1536 mutated in this work. (f) Products from mixing Lhr‐CTD or Lhr‐CTD^D1536A^ proteins (50 nM) with 12.5 mM d‐uracil containing 5′ Cy5‐ssDNA substrate, viewed in a 18% acrylamide denaturing TBE gel. Product formation is shown every 5 min for 30 min, observing no glycosylase activity from Lhr‐CTD^D1536A^.

We investigated whether *E. coli* Lhr is capable of DNA glycosylase activity, as suggested from structural similarities between glycosylases and the uncharacterized C‐terminal region of *E. coli* Lhr (Lhr‐CTD, LHR amino acids 876–1538) (Buckley et al., 2020; Warren et al., 2021) (Figure 1a). *E. coli* Lhr‐CTD protein fragment and full‐length Lhr protein (1538 amino acid) were purified (Figure 1b)—when Lhr‐CTD (50–800 nM) was mixed with a Cy5‐end labeled 37‐nt ssDNA molecule that was modified to contain a single uracil nucleotide located 18 nucleotides from the 5′ ssDNA end (uracil‐ssDNA) a single product was observed on alkaline treatment of reactions (Figure 1c, compare lanes 1–6 with 7–12), indicating DNA strand breakage at an abasic site consistent with glycosylase activity. No product was observed from Lhr‐CTD mixed with the same ssDNA lacking chemical modification (Figure 1d ).

**FIGURE 2:**
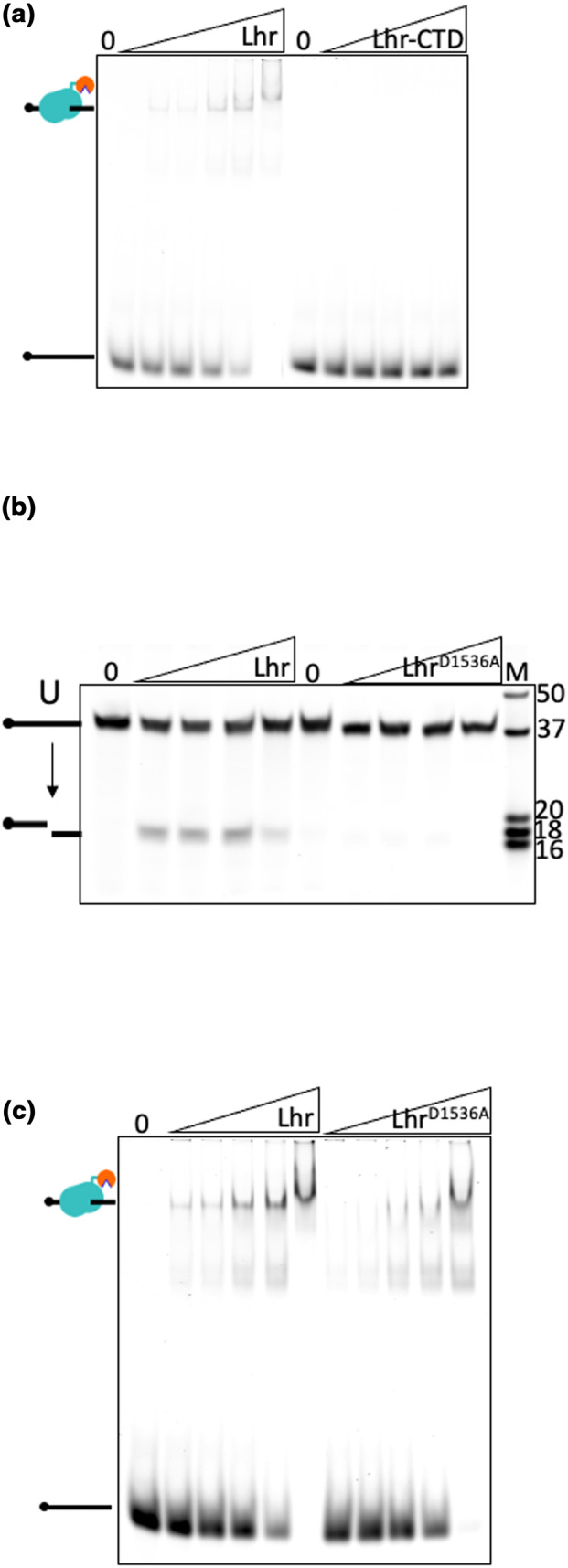
Lhr^D1536A^ is inactive as a glycosylase but binds to DNA. (a) EMSA assays showing Lhr (12.5, 25, 50, 100, and 200 nM) complexes bound to single‐stranded DNA (12.5 nM, Supporting Information Table S2) that are stable migrating through a 5% acrylamide TBE gel (indicated to left of the gel as protein bound to ssDNA), compared with Lhr‐CTD at the same concentrations. (b) Products of Lhr glycosylase activity seen in an 18% acrylamide denaturing TBE gel were absent when reactions contained Lhr^D1536A^. Proteins were used at 25, 50, 100, and 200 nM, with 12.5 nM of d‐uracil containing 5′ Cy5‐ssDNA substrate. (c) EMSA showing that Lhr^D1536A^ and Lhr (12.5, 25, 50, 100, and 200 nM) form stable complex with Cy5 end‐labeled single‐stranded DNA (Supporting Information Table S2) in a 5% acrylamide TBE gel.

To validate the UDG activity of *E. coli* Lhr‐CTD, we sought to identify single amino acid substitutions that would inactivate it. Sequence alignment of *E. coli* Lhr‐CTD and AlkZ, with which it has structural similarity (Buckley et al., 2020; Warren et al., 2021), were unproductive at identifying highly conserved residues because Lhr‐CTD lacks the ‘QxQ’ motif characteristic of AlkZ protein active sites (Mullins et al., 2017), therefore suggesting an alternate catalytic mechanism in Lhr. We instead identified potential active site amino acids through visual scrutiny of the Phyre2 (Kelley et al., 2015) predicted model of the *E. coli* Lhr‐CTD, and in particular the positioning of side chains proximal to a proposed glycosylase active site (Figure 1e). Purified Lhr‐CTD^D1536A^ gave no glycosylase product when mixed with the uracil‐modified ssDNA, compared with Lhr‐CTD (Figure 1f). We then tested whether substitution of the Lhr Asp‐1536 residue inactivated UDG active site chemistry or had some other effect on the protein that perturbed DNA binding. Unmutated Lhr‐CTD was unable to form stable complex with a single‐stranded DNA molecule (Supporting Information Table S2) in electrophoretic mobility shift assays (EMSAs) that is bound by full‐length Lhr (Figure 2a). Therefore, we purified and tested full‐length Lhr^D1536A^. Lhr was also active as an UDG compared with Lhr‐CTD (Figure 2b), but the Lhr^D1536A^ mutation inactivated glycosylase activity in agreement with inactive Lhr‐CTD^D1536A^ fragment (Figure 2c). In EMSAs, Lhr^D1536A^ formed stable complex with DNA similarly to Lhr (Figure 2d); therefore, we conclude that Lhr is a UDG that requires an active site aspartic acid residue.

### 2.2 DNA glycosylase activity of *E. coli* Lhr is independent from its DNA helicase activity

**FIGURE 3:**
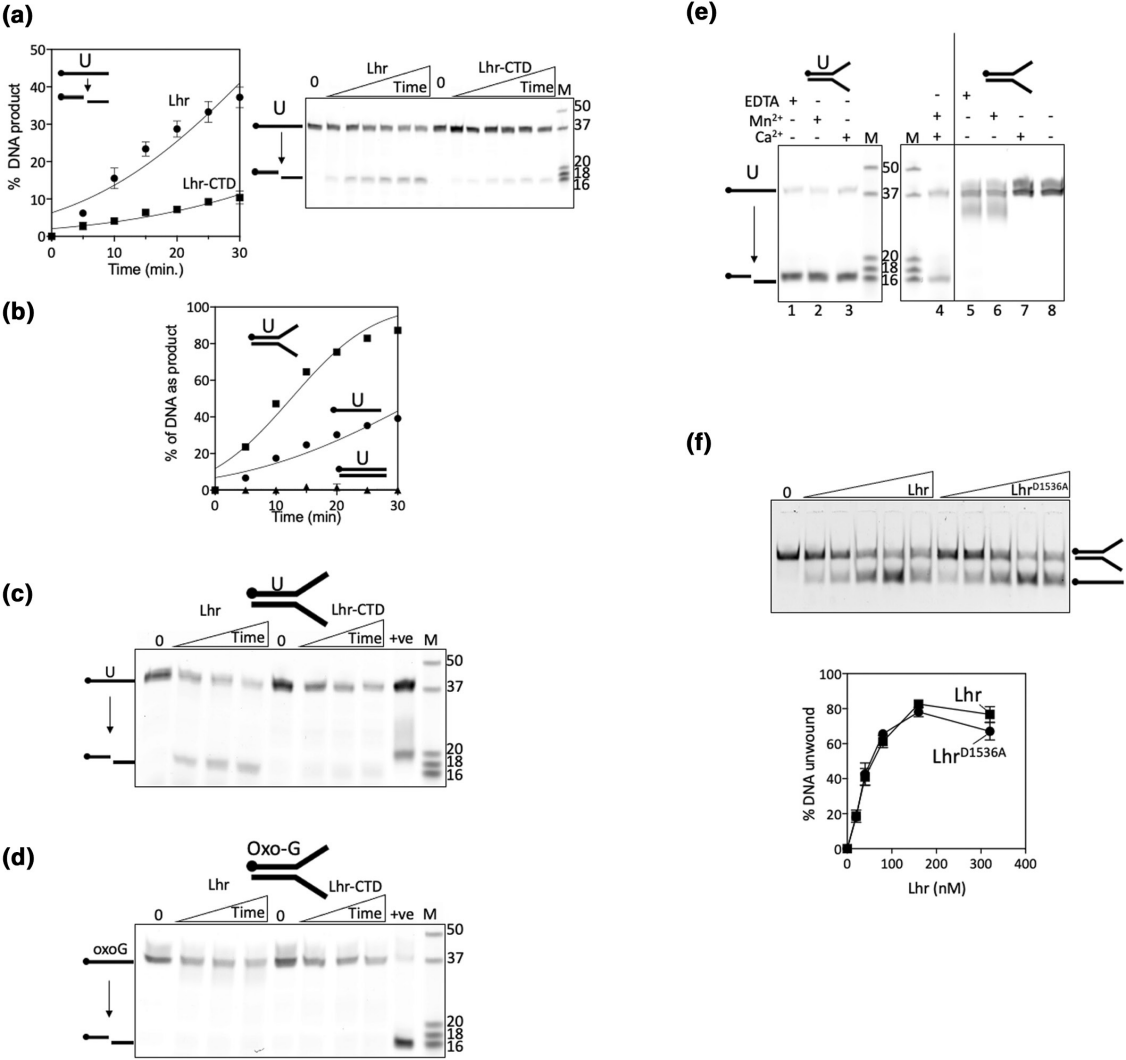
Lhr is inactive against 8‐oxoguanine, and its uracil‐DNA glycosylase activity on duplex DNA functions independently from Lhr helicase activity. (a) Time‐dependent uracil‐DNA glycosylase activity of Lhr (50 nM) compared with Lhr‐CTD. The data show means of glycosylase activity (*n* = 3, with bars for standard error) alongside a representative gel that was used for quantification. The cartoon to the top left of the graph indicates Lhr‐catalyzed hydrolysis of the uracil‐containing ssDNA, as shown in gels in Figure 1. (b) Comparison of Lhr (50 nM) glycosylase activity on ss‐, ds‐, and forked d‐uracil containing DNA substrates (12.5 nM) as a function of time, with samples taken at time points indicated—plots are means of two independent experiments showing standard error bars. (c) Time‐course assays (10, 20, and 30 min) showing products from Lhr and Lhr‐CTD (each 80 nM) mixed with the preferred flayed duplex uracil‐DNA, seen in an 18% acrylamide denaturing TBE gel. Known length DNA strands are shown (M) and the positive control reaction (+ve) is product from 5 units of *E. coli* uracil‐DNA glycosylase. (d) As for (c) except d‐uracil‐DNA was replaced with otherwise identical 8‐oxo‐d‐Guanine DNA, and the control reaction (+ve) shows product formed by 5 units of formamidopyrimidine DNA glycosylase (Fpg) protein. (e) Lhr (80 nM) uracil‐DNA glycosylase activity seen as products in 18% acrylamide denaturing TBE gels (lanes 1–4), after 30‐minute reactions in either EDTA, manganese or calcium, each replacing magnesium as indicated, compared with unmodified DNA (lanes 5–8). (f) Shows a representative gel and graphical plot of DNA unwinding by Lhr and Lhr^D1536A^ proteins (20, 40, 80, 160, and 320 nM) on 12.5 nM of 5′ Cy5 labeled flayed duplex DNA, assessed in 10% acrylamide TBE gels. The data plot shows means of three experiments for each protein, showing standard error bars.

Full‐length Lhr was substantially more active than Lhr‐CTD as a UDG when measured in assays as a function of time (Figure 3a)—this may be explained by much more stable DNA binding by full‐length Lhr compared with Lhr‐CTD that was observed in EMSAs (Figure 2a). We therefore continued to use full‐length Lhr to further investigate UDG function against flayed duplex DNA molecules that are substrates for unwinding by the Lhr 3′ to 5′ DNA helicase activity (Buckley et al., 2020). For this work, the duplex substrate was formed from annealing uracil‐containing ssDNA with its unmodified complementary DNA strand, with uracil positioned 8 nt from the fork branchpoint, 18 nt from the Cy5‐DNA 5′ end. Measured as a function of time, Lhr generated glycosylase product from the uracil duplex at least fivefold more effectively than when incubated with uracil‐ssDNA (Figure 3b), and Lhr was more active than Lhr‐CTD on the uracil‐fork DNA (Figure 3c). Neither Lhr nor Lhr‐CTD gave any glycosylase product when uracil was substituted for a single 8‐oxoguanine residue at the same position in DNA (Figure 3d). The product of Lhr from uracil‐DNA single strands or duplex migrated close to the 16 nt marker, indicating that the same glycosylase product was formed from both substrates. Glycosylase assay conditions in Figures 1, 2, 3 included Mg^2+^ in the reaction buffer, but Lhr was also active in buffers containing EDTA, Mn^2+^ and Ca^2+^ instead of Mg^2+^ (Figure 3e lanes 1–4) but was inactive as a glycosylase on DNA lacking a uracil residue (Figure 3e lanes 5–8). Lhr^D1536^ that is inactive as a UDG was proficient at fork DNA unwinding comparably to wild‐type Lhr protein (Figure 3f), indicating functional separation of helicase and uracil glycosylase catalytic activities, but we observe that glycosylase activity is enhanced when the helicase domains are present and contribute to DNA binding (Figure 2a).

### 2.3 *E. coli* cells lacking LHR are sensitive to oxidative stress

**FIGURE 4:**
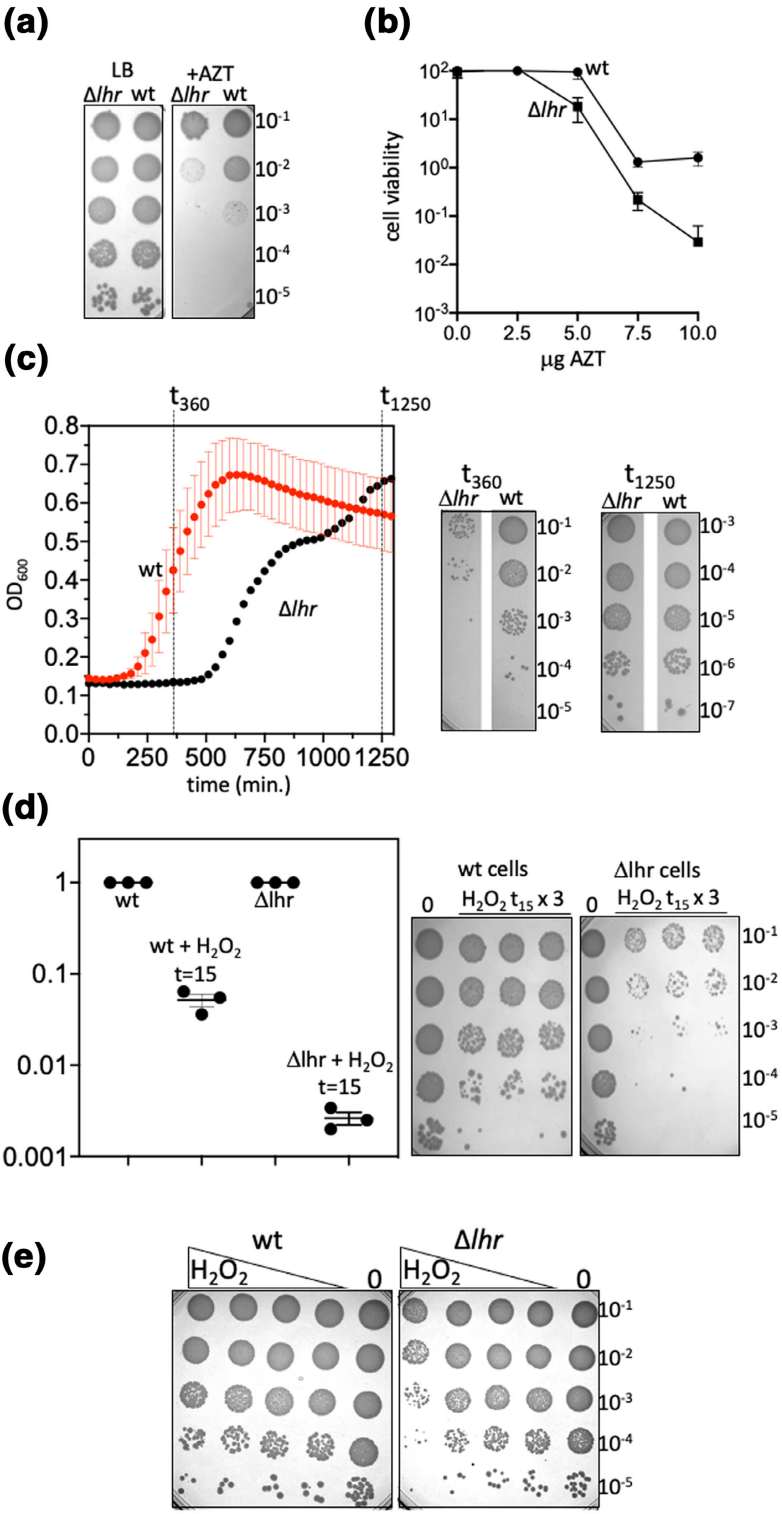
*E. coli* cells lacking Lhr are sensitive to oxidative stress. (a) Viability spot tests showing moderately increased sensitivity of Δ*lhr* cells to AZT (7.5 μg/mL) compared with wild‐type (wt) cells, and; (b) represented in viability curves when Δ*lhr* and wild‐type cells were grown independently in media containing AZT at 2.5, 5, 7.5, or 10 μg/mL. The plots show grow relative to wild‐type cells grown in media lacking AZT, as means of three experiments. (c) Growth of Δ*lhr* and wild‐type cells monitored in 96‐well plates by optical density in media containing 5.8 mM H_2_O_2_, and with corresponding representative viability spot tests taken at the time points indicted during growth. (d) Viability of Δ*lhr* and wild‐type cells in response to 15 min (t_15_) exposure to hydrogen peroxide. Growths were in triplicate, with a mean of three colony counts from wild‐type and Δ*lhr* cells that were untreated, in the graph represented as 3 data points all at 1.0 as mean, and on the agar plates represented in the lane marked 0. Data points for colony counts after t_15_ hydrogen peroxide were calculated from the mean (1.0) untreated cultures and are shown as the three actual data values with standard error bars. The agar plates highlight the difference between wild‐type and Δ*lhr* cells. (e) Viability spot tests comparing Δ*lhr* and wild‐type cells grown without H_2_O_2_ to optical density prior to plating on to LB media containing 1.5625, 3.75, 6.25, and 12.5 mM H_2_O_2_.

Oxidative damage to DNA in *E. coli* cells includes chemical changes to DNA that result in deamination of cytosine to uracil or oxidized uracil derivatives (Almatarneh et al., 2008; Cadet & Wagner, 2013; Kreutzer & Essigmann, 1998; Krokan et al., 2002), potentially triggering cytosine to adenine transversion mutations. We assessed for a contribution from Lhr to repair of oxidative DNA damage in *E. coli* cells that may be consistent with its in vitro UDG activity. The *lhr* gene was deleted in *E. coli* MG1655 (Δ*lhr*) by recombineering, and we removed the inactivating antibiotic resistance marker, verified by sequencing across the deletion site. We first tested Δ*lhr* cells for sensitivity to AZT, a previously reported phenotype for Lhr in *E. coli* cells (Cooper et al., 2015). In a viability plate assay after growing cells in broth (LB) containing a fixed concentration of 7.5 μg/mL AZT, we observed 10‐fold reduced viability of Δ*lhr* cells compared with wild‐type cells (Figure 4a), and similarly moderate sensitivity of Δ*lhr* cells across AZT concentrations (Figure 4b), agreeing with the previous study (Cooper et al., 2015). To test for a phenotype from oxidative damage, we first measured cell survival when grown in media containing hydrogen peroxide. Hydrogen peroxide (5.8 mM) added to growth media resulted in significantly reduced optical density of Δ*lhr* cells in exponential phase compared with wild‐type cells, which corresponded with at least 100‐fold less Δ*lhr* cell numbers at the 360‐minute time point (Figure 4c). This indicated that Δ*lhr* cell populations are more slowly established than wild‐type cells under these oxidative stress conditions, but we also observed that these cells recover to full viability after 1000+ min, shown at 1250 min (Figure 4c). This sensitivity of growing Δ*lhr* cells to hydrogen peroxide was consistent with substantially reduced viability of Δ*lhr* cells (10–100‐fold) compared to wild‐type cells within 15 min of adding hydrogen peroxide to growth media in which both were similarly viable beforehand at OD 0.4 (Figure 4d). Finally, when similarly viable untreated wild‐type and Δ*lhr* cells were spotted onto LB agar containing increasing concentrations of hydrogen peroxide Δ*lhr* cells showed 10–100‐fold reduced viability (Figure 4e). In contrast, Δ*lhr* cells we not sensitive to the DNA cross‐linking agent mitomycin C (Supporting Information Figure S1), consistent with overcoming oxidative DNA damage rather than DNA cross‐links, which are removed by bacterial AlkZ family glycosylases, which had been predicted to share functional similarity with Lhr (Buckley et al., 2020).

## 3 DISCUSSION

We provide evidence that *E. coli* Lhr is a UDG (Lhr‐UDG), a new function for bacterial Lhr proteins alongside their well‐characterized 3′ to 5′ single DNA translocation activity that is stimulated by forked or flayed DNA substrates (Buckley et al., 2020; Ejaz & Shuman, 2018; Ordonez & Shuman, 2013). We show that the Lhr‐UDG activity requires an active site aspartate residue (Asp‐1536), similarly to the active site aspartate general base (Asp‐62) that is essential for major groups of UNG/UDG proteins (Aravind & Koonin, 2000). The Lhr UDG function is positioned in the previously uncharacterized Lhr‐CTD—though this fragment of Lhr was proficient as a ‘stand‐alone’ UDG, its activity was significantly increased by the presence of the Lhr helicase domains, by the helicase domains providing more stable DNA binding compared with Lhr‐CTD. Inactivating the Lhr‐UDG activity did not inactivate DNA unwinding by Lhr, providing further support for the DNA binding functions of Lhr being concentrated in the helicase domains.

The different active site residues of Lhr and the AlkZ family of glycosylases—Lhr lacks the QxQ motif characteristic of AlkZ—suggests distinct DNA repair functions, supported from our observation that Δ*lhr* cells show no sensitivity to the DNA cross‐linker mitomycin, which is overcome by AlkZ proteins. In agreement with a previous study (Cooper et al., 2015), we observed loss of Lhr from bacterial cells (Δ*lhr*) caused mild sensitivity to the DNA polymerase inhibitor AZT, a phenotype we also observed from our independently generated Δ*lhr* cells and removal of the inserted antibiotic resistance marker. We identified that Δ*lhr* cells were significantly more sensitive than wild‐type cells to oxidative stress induced by hydrogen peroxide, for example, showing rapidly reduced cell viability on treatment with hydrogen peroxide consistent with a role in DNA repair (Carlsson & Carpenter, 1980). Hydrogen peroxide treatment is one of several routes causing genetic damage by cytosine deamination in bacterial cells and is consistent with our observations of Lhr‐UDG robustly removing uracil from DNA in vitro but being inactive against 8‐oxoguanine. However, we cannot rule out that Lhr in cells is targeted to other chemically modified bases, perhaps instead of or in addition to uracil, which are prevalent during oxidative stress, including thymine‐glycols and hydro‐uracils or—thymines. It is clear from previous studies in archaea and bacteria that Lhr binds to single‐stranded DNA, triggering its ATP‐dependent DNA translocase/helicase activity. This suggests that Lhr identifies and removes bases such as uracil in the context of single‐stranded DNA (perhaps during replication or transcription) rather than in nucleotide pools and supported by the truncated Lhr‐CTD showing much reduced DNA binding activity and much less UDG activity than full Lhr (Figure 3).

UDGs are ubiquitous in nature, although this is the first report of a UDG fused to a DNA helicase. *E. coli* has a canonical UDG enzyme that functions in global DNA repair coupled with stable DNA replication—upregulation of mycobacterial Lhr in response to mitomycin C treatment (Rand et al., 2003), and the sensitivity of *E. coli* cells to the polymerase inhibitor AZT when they lack *lhr*, may indicate that Lhr is activated for repair of oxidative DNA damage in specific contexts requiring DNA replication, for example, during homologous recombination, or during transcription, rather than for genome‐wide reconnaissance typified by *E. coli* UNG enzyme. In this context, removal of uracil from DNA by Lhr may protect genetic fidelity at sites that are overcoming blocked DNA replication or transcription. We did not observe any evidence that *E. coli* cells lacking Lhr are susceptible to hypermutation when grown under standard conditions in nutrient broth, without any exogenous oxidative stress (Figure S2), which we suggest may also be consistent with Lhr providing a protective effect against uracil mutagenesis under specific physiological conditions. We conclude that Lhr is a DNA helicase and glycosylase, which forms products more similar in our in vitro reactions to the glycosylase‐AP‐lyase Fpg control protein, rather than the glycosylase UDG control reactions (Figure 3), although preferring uracil not 8‐oxoguanine.

## 4 EXPERIMENTAL PROCEDURES

### 4.1 Proteins

DNA sequences of primers and substrates, plasmids, and *E. coli* strains are detailed in Supplementary data. *E. coli* MG1655 gene b1653, encoding Lhr, was PCR‐amplified from genomic DNA, and cloned into pT7‐7 using *Nde*I and *Hin*DIII restriction sites generating pRJB28 for expression of hexa‐histidine tagged Lhr. Lhr‐CTD DNA encoding Lhr amino acids 876–1538 was amplified and cloned by ligation‐independent cloning (LIC) into the Bsa I site of pNH‐TrxT (GU269914.1 [Savitsky et al., 2010]), a vector based on pET28a for effective overexpression of proteins in *E. coli*. These plasmids were used to generate Lhr^D1536A^ using mutagenic primers in PCR by Q5 hot start polymerase, and resulting reactions were treated with *Dpn*I, T4 polynucleotide kinase, and DNA ligase. Plasmid DNA was extracted and sequenced from colonies after transforming reaction mixtures into *E. coli*.

Lhr and C‐Lhr proteins were overexpressed in *E. coli* Rosetta 2 cells grown in MU broth containing ampicillin and chloramphenicol (pT7‐7) or kanamycin and chloramphenicol (pNH‐TrxT). Cells were grown with shaking at 37°C to OD_600_ of 1.2 and transferred to an ice slurry for cooling before addition of IPTG (0.8 mM). Growth was continued for 10 h at 18°C, and cells were harvested and resuspended in 20 mM HEPES pH 8.0, 1.5 M ammonium sulfate, 20 mM imidazole, and 10% (w/v) glycerol (Ni‐NTA buffer A) containing 0.1 mM phenylmethylsulfonyl fluoride (PMSF). This process and purification was also followed for obtaining Lhr^D1536A^ protein. Cells were thawed and sonicated on ice, clarified by centrifugation, and soluble proteins were loaded into a 5‐mL butyl sepharose column equilibrated with 20 mM HEPES pH 8.0, 1.5 M ammonium sulfate, and 10% (w/v) glycerol (hydrophobic salt buffer A). The column was washed with 20 mM HEPES pH 8.0 and 900 mM ammonium sulfate. Then, a 5‐mL Ni‐NTA column pre‐equilibrated with Ni‐NTA buffer A was attached in tandem with the butyl sepharose column and columns washed with 20 mM HEPES pH 8.0 and 10% glycerol until no proteins were detectable by UV monitoring as eluting from the columns. The butyl sepharose column was removed, and Lhr was eluted from the Ni‐NTA column by increasing imidazole to 500 mM in 20 mM HEPES and 10% glycerol. Lhr‐containing fractions were pooled and dialyzed overnight into 20 mM HEPES pH 8.0 and 150 mM NaCl, 10% (w/v) glycerol (low salt buffer A) and loaded into a 1 mL of Q‐sepharose column. Lhr eluted in an increasing gradient of NaCl to 1.5 M. Lhr fractions were pooled and dialyzed overnight for storing in 20 mM HEPES pH 8.0, 150 mM NaCl and 35% (w/v) glycerol for aliquoting, flash freezing, and storage at −80°C. *E. coli* UDG and formamidopyrimidine DNA glycosylase (Fpg) control proteins (Figure 3) were purchased from New England Biolabs.

### 4.2 In vitro DNA binding, unwinding and glycosylase assays

DNA strands for substrate formation (Supplemental data) were synthesized with Cy5 end label. DNA binding was assessed using EMSAs. Reactions were incubated at 37°C for 20 min in helicase buffer (HB), 20 mM Tris pH 7.5, 10% (v/v) glycerol, 100 μg/mL BSA, using 12.5 nM Cy5‐fluorescently labeled DNA substrate, 25 mM DTT and 5 mM EDTA and then placed on to ice for 10 min. Orange G and 80% (v/v) glycerol (OG) was added to load reactions onto a 5% acrylamide TBE gel that was electrophoresed for 1 h 30 min at 140 V. Gels were imaged using a Typhoon phosphor‐imager (Amersham) at 633 nm using a R765 filter for Cy5 detection.

DNA unwinding assays were at 37°C in reactions containing buffer HB, 12.5 nM Cy5‐fluorescently labeled DNA substrate, 25 mM DTT, 1.25 μM unlabeled ‘trap’ DNA, 5 mM ATP, and 5 mM CaCl_2_. Reactions were pre‐incubated at 37°C for 5 min without the ‘trap’ or ATP before they were added together to start the reactions for 30 min at 37°C, stopped by addition of stock stop solution (4 μL per 20 μL reaction); 2 mg/mL proteinase K in 200 mM EDTA and 2.5% (w/v) SDS. OG dye was added for electrophoresis through a 10% acrylamide TBE gel for 45 min at 150 V. Gels were imaged using a Typhoon phosphor‐imager (Amersham) at 633 nm using a R765 filter for Cy5 detection.

DNA glycosylase reactions were at 37°C in reaction mixtures containing buffer HB, 12.5 nM Cy5‐fluorescently labeled DNA substrate, 25 mM DTT, 5 mM ATP, 4 mM MnCl_2_, and 4 mM CaCl_2_. Reactions were pre‐incubated at 37°C before being initiated by addition of Lhr protein and (unless in a time course assay) allowed to continue for 30 min before addition of stock stop solution and 4 μL of 1 M NaOH. Reaction samples were boiled for 5 min and formamide added before loading into a 15% denaturing (8 M urea) acrylamide TBE gel for 4 h at 5 watts per gel. Gels were imaged using a Typhoon phosphor‐imager (Amersham) at 633 nm using a R765 filter for Cy5 detection, generating TIFFs that were measured using Gel Analyzer 19.1 (Lazar) software. Graphs of glycosylase activity were generated using prism (GraphPad).

### 4.3 Generation of a chromosomal deletion of *E. coli lhr*

DNA constructs and strain genotypes are presented in the Supplementary material. *Lhr* deletion was by recombineering (Datsenko & Wanner, 2000) and P1 transduction of an FRT (FLP recognition target) flanked Kan^r^ marker. To generate an effective P1 stock, the overnight culture was used to inoculate 8 mL of Mu broth containing 6 mM CaCl_2_. A sample of the cells (0.1 mL) grown at 37°C to OD_600_ 0.8–1.0 in a shaking water bath was added to four overlay tubes each containing 3 mL of 0.4% w/v Mu broth agar held at 42°C. P1 phage stock was diluted 10–100‐fold in MC buffer (100 mM MgSO_4_, 5 mM CaCl_2_) and 0.05 mL, 0.1 mL or 0.2 mL of this diluted phage was added to the overlay tubes containing cells and molten agar and gently mixed. The remaining tube was left without phage as a control. The contents of each overlay tube was poured onto P1 agar plates and left to set for overnight growth at 37°C for 18 h. Soft agar from phage‐lysed plates was added to 1 mL of MC buffer (100 mM MgSO_4_ and 5 mM CaCl_2_) and 0.5 mL of chloroform for vigorous mixing before centrifugation at 5752 rcf for 20 min at 4°C. The supernatant was retrieved and mixed with chloroform (0.5 mL) for storage at 4°C as a P1 phage stock. MG1655 recipient strain was grown in a Mu broth to OD_600_ 0.8 using a shaking water bath. Cells were pelleted, resuspended in 1 mL of MC buffer, and left at 25°C for 10 min. 0.2 mL of cells were added into three overlay tubes containing 0 mL, 0.05 mL, and 0.2 mL of P1 lysate produced previously and incubated for 30 min at 37°C. Cell/P1 lysate mix was added to 2.5 mL of 0.6% agar, mixed gently, and poured onto Mu broth agar plates containing 30 μg/mL kanamycin and left to set. Plates were grown for 1–2 days, lid‐up, at 37°C to allow colonies to develop. Colonies were then picked and purified by streaking onto Mu broth agar plates containing no antibiotic. This was repeated three times before plating again onto agar containing 30 μg/mL kanamycin for confirmation of gene knockout and Kan^r^‐FTR insertion.

Successful P1‐treated MG1655 cells were transformed with pCP20. Transformants were picked and used to inoculate 8 mL of Mu broth containing no antibiotic. Culture was grown overnight at 45°C in a shaking water bath FLP recombinase expression and plasmid curation. Cells were then streaked onto Mu broth agar plates to produce single colonies and grown at 37°C overnight. Colonies were restreaked three times before replica plating onto Mu broth agar plates containing 50 μg/mL ampicillin, 30 μg/mL kanamycin and then no antibiotic to confirm loss of the pCP20 plasmid. Isolates which only grew on the no antibiotic agar plates were grown overnight for glycerol stock production and streaked a further time for colony PCR diagnostic confirmation.

### 4.4 *E. coli* viability spot assays

Cell viabilities were measured from liquid cultures grown to OD_600_ 0.3–0.4 in a shaking water bath at 37°C monitored in the growth tubes by using a Spectronic 20+. Cells were then treated by addition to the growth media of hydrogen peroxide (H_2_O_2_) or AZT at concentrations stated in the results. Cells were grown for a further 30 min and then serially diluted into 1× M9 medium to arrest growth for spotting (10 ul) on to agar plates grown overnight in a 37°C incubator. For comparing growth curves cells were grown to OD_600_ 0.3–0.4 and then transferred into a 24‐well flat‐bottomed plate, and H_2_O_2_ was added to appropriate wells to the given concentration from a 0.98 M stock. Growth in the plates was monitored with orbital shaking in a FLUOstar microplate reader (BMG Labtech). OD_600_ readings were taken every 30 min in this time, and data were extracted and analyzed using Prism (GraphPad) software.

## AUTHOR CONTRIBUTIONS

Edward L. Bolt and Christopher D. O. Cooper designed the project and with RJB wrote the paper. Ryan J. Buckley, Anna Lou‐Hing, Karl M. Hanson, Nadia R. Ahmed, and Christopher D. O. Cooper performed experiments, analyzed data, and generated images.

## ETHICS STATEMENT

This study does not involve human subjects, patient medical records or animals.

## Supporting information


Figure S1.

## Data Availability

The data that support the findings of this study are available from the corresponding author upon reasonable request.
