# Supplementary material for: Escherichia coli DNA repair helicase Lhr is also a uracil‐DNA glycosylase
Source: Mol Microbiol. 2023 Jul 14;120(2):298–306. doi: 10.1111/mmi.15123 (PMC10953399; doi:10.1111/mmi.15123)

**Supplementary File Table S1: *E. coli* strains**

| DH5α | New England BioLabs | F– Φ80*lacZΔ*M15 Δ(*lac*ZYA*- arg*F)U169 *rec*A1 *end*A1 *hsd*R17 (rK–, mK+) *pho*A *sup*E44 *thi*- 1 *gyr*A96 *rel*A1 λ*–* |
| --- | --- | --- |
| BL21 AI | Thermofisher | F– *ompT hsdS*B (rB-mB-) *gal dcm araB*::*T7RNAP-tetA* |
| MG1655 | Reference [1] |  |
| RB001a | This work:  *Δlhr* KanR | P1 transduction from Keio collection ‘CGSC# - 9400’ into MG1655. |
| RB002a | This work:  *Δlhr* | RB001a pCP20 treated to remove KanR FRT scar. |

1. Blattner, F.R., et al., *The complete genome sequence of Escherichia coli K-12.* Science, 1997. **277**(5331): p. 1453-62.

**Supplementary File Table S2 – DNA Substrates:** The blue circle in each case denotes the position of the Cy5 moiety.

| Name |  | Sequence and modifications of DNA oligonucleotides (5’ to 3’) |
| --- | --- | --- |
| Flayed duplex DNA (EMSA & helicase assays) | 1 | **●**TCGGATCCTCTAGACAGCTCCATGATCACTGGCACTGGTAGAATTCGGC |
|  | 2 | CAACGTCATAGACGATTACATTGCTACATGGAGCTGTCTAGAGGATCCGA |
| DNA-MW12-37mer | 1 | **●**GTCGGATCCTCTAGACAGGCTCCATGCGTAGTACTCG |
| d-U DNA | 1 | **●**GTCGGATCCTCTAGACA**U**GCTCCATGCGTAGTACTCG |
| oxo-d-G DNA | 1 | **●**GTCGGATCCTCTAGACA**G**GCTCCATGCGTAGTACTCG |
| d-U duplex | 1 | d-U DNA |
|  | 2 | CGAGTACTACGCATGGAGCGTGTCTAGAGGATCCGAC |
| DNA fork | 1 | **●**GTCGGATCCTCTAGACACGCTCCATGTAGCAATGTAAT |
|  | 2 | ATTACATTGCTACATGGAGCGTGTCTAGAGGATCCGAC |
| d-U fork | 1 | d-U DNA |
|  | 2 | ATTACATTGCTACATGGAGCCTGTCTAGAGGATCCGAC |
| oxo-d-G fork | 1 | oxo-d-G DNA |
|  | 2 | ATTACATTGCTACATGGAGCGTGTCTAGAGGATCCGAC |

**Supplementary Figure S1:** Viability of Δ*lhr* and wild type cells grown in media containing mitomycin at 0.2, 0.5, 1.0 μg/mL. The growth method used was the same as that for hydrogen peroxide, described in the main methods. The plots are means of two independent experiments, showing standard error from the means, with panels below showing colonies from a representative single experiment.


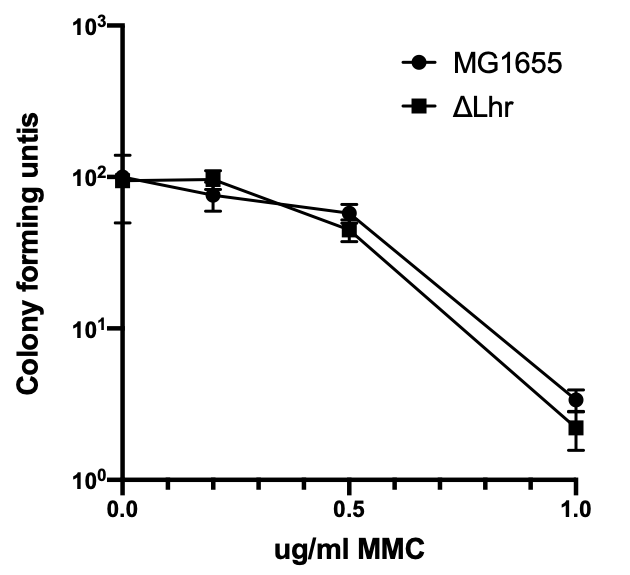


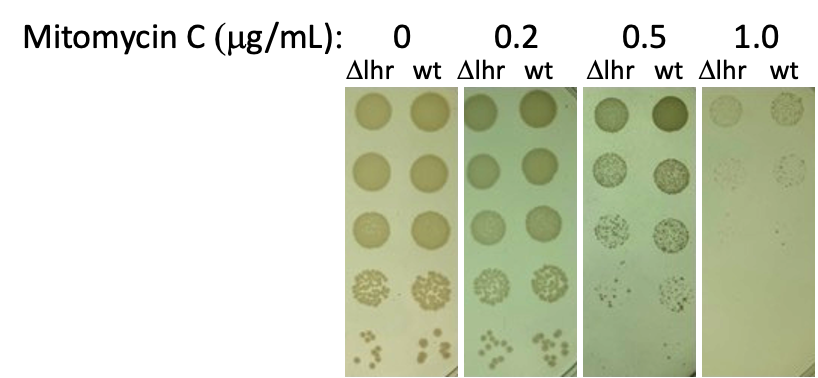


**Supplementary Figure S2:** Liquid cultures of *E. coli* MG1655 or MG1655 Δlhr were grown in LB media (8 mL) with shaking as described in the main methods, to O.D. readings of 0.4, 0.6, or for 24 hours (1 day) or 48 hours (2 days), as indicated below. Cells (100 μl) were then spread on to LB agar, or LB agar containing rifampicin at 10 or 20 μg/mL to detect any hyper-mutation phenotype.


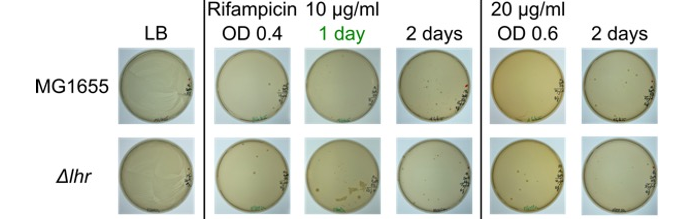

Supplement: Supplementary file 1 — Figure S1. [file MMI-120-298-s001.docx]
